# A Segmented Cross-Correlation Algorithm for Dynamic North Finding Using Fiber Optic Gyroscopes

**DOI:** 10.3390/s24020322

**Published:** 2024-01-05

**Authors:** Shuwei Fang, Shanjun Mao, Yanjun Chen, Lanxin Zhu

**Affiliations:** 1 Institute of Remote Sensing and Geographic Information System, Peking University, Beijing 100871, China; fangshuwei@pku.edu.cn; 2 State Key Laboratory of Advanced Optical Communication Systems and Networks, School of Electronics, Peking University, Beijing 100871, China; chenyanjun@pku.edu.cn (Y.C.); zhulanxin2020@stu.pku.edu.cn (L.Z.)

**Keywords:** north finding, fiber optic gyroscope, divide-and-conquer algorithm, jitter

## Abstract

Fiber optic gyroscope (FOG)-based north finding is extensively applied in navigation, positioning, and various fields. In dynamic north finding, an accelerated turntable speed shortens the time required for north finding, resulting in a rapid north-finding response. However, with an increase in turntable speed, the turntable’s jitter contributes to signal contamination in the FOG, leading to a deterioration in north-finding accuracy. This paper introduces a divide-and-conquer algorithm, the segmented cross-correlation algorithm, designed to mitigate the impact of turntable speed jitter. A model for north-finding error is established and analyzed, incorporating FOG’s self-noise and the turntable’s speed jitter. To validate the feasibility of our method, we implemented the algorithm on a FOG. The simulation and experimental results exhibited a strong concordance, affirming the validity of our proposed north-finding error model. The experimental findings indicate that, at a turntable speed of 180°/s, the north-finding bias error within a 360 s duration is 0.052°, representing a 64% improvement over the traditional algorithm. These results indicate the effectiveness of the proposed algorithm in mitigating the impact of unstable turntable speeds, offering a solution for north finding with both prompt response and enhanced accuracy.

## 1. Introduction

Directional information is crucial for establishing coordinate systems in engineering and indicating relative positions in geographic space. North finding, a technique employed to detect and calculate azimuth [1,2], plays a pivotal role in this context. Various methods exist for north-finding [3,4,5], with the inertial north-finding approach being the most widely utilized. This method leverages inertial instruments, such as gyroscopes, to observe the Earth’s rotational projection. Algorithms are then employed to determine the azimuth with respect to the geographic north. The current demand for north finders not only demonstrates high precision but also facilitates prompt azimuth determination [6].

Despite the high orientating precision of gyro-theodolite, based on the appended mechanical gyroscope, it is burdened with limitations, such as high cost, complex structure, prolonged orientating time, and substantial drift [7,8]. The fiber optic gyroscope (FOG), on the other hand, has emerged as a commonly used instrument for inertial north-finding [9,10]. Depending on the FOG’s motion state, north-finding methods can be classified into static north-finding and dynamic north-finding methods [11]. The static method typically involves utilizing a turntable to position the gyroscope at multiple sampling locations and statically measuring the Earth’s rotation rate projection at these positions [12,13,14,15,16,17,18]. However, introducing multiple measurement positions while enhancing accuracy imposes a significant time burden and requires an exact turntable positioning [19].

Numerous studies have focused on dynamic north-finding methods to expedite and enhance the accuracy of north-finding [19,20,21,22,23]. The dynamic method involves the continuous rotation of the gyroscope, driven by the turntable at a predetermined speed, with the azimuth angle determined by analyzing the continuous gyroscope output signal [20,21,24,25]. The robustness of the dynamic method to bias error, scale factor deviation, and temperature drift has been demonstrated in the literature [21,22,26] and compared to static north-finding. The majority of research on dynamic north finding has focused on lower rotation speeds, usually less than 60°/s [19,21,22]. This is because, as turntable speed increases, both north-finding variance and bias errors escalate significantly, challenging the effectiveness of north-finding at higher speeds and impeding efforts to enhance accuracy [19,21].

The jitter effect of turntable speed is a significant factor affecting north-finding accuracy at high speeds [21,22]. Commonly used dynamic north-finding methods employ least squares [8,23] and frequency cross-correlation methods [21,22,27] in the azimuth calculation, assuming that the signal obtained is a sinusoidal function with a fixed frequency. However, the FOG’s output signal frequency can be distorted due to random speed jitter effects caused by environmental vibrations or servo motors. This effect can be mitigated by employing a band-pass filter matching the rotating frequency [21]. Nevertheless, errors in this method at high speeds remain significant due to the actual turntable speed deviations from the ideal value.

Considering the current demand for short-duration, high-precision north finding and the challenge of improving dynamic north-finding accuracy at high speeds, this paper proposes a dynamic north-finding algorithm based on segmented cross-correlation (DNF-SCC). As speed jitter is randomly distributed throughout north finding, our algorithm employs a divide-and-conquer approach by segmenting FOG output data to suppress its effect. For each segment, the signal frequency analysis and azimuth angle calculation are performed separately, mitigating the impact of the turntable speed jitter on the measurements. The simulation and practical experimental results demonstrate that, compared with existing algorithms, the DNF-SCC algorithm significantly improves north-finding accuracy at high turntable speeds. For example, at a turntable speed of 180°/s, the traditional algorithm yields a north-finding bias error of 0.145° after 360 s, while the proposed algorithm achieves an error of 0.052°, representing a 64% improvement in accuracy. These results highlight the application potential of the DNF-SCC algorithm, particularly in scenarios requiring rapid and high-precision north finding.

The remaining sections of this paper are organized as follows. Section 2 introduces the principles and algorithms of the DNF-SCC algorithm and describes an analysis model for the impact of FOG noise and turntable speed jitter on north finding at high speeds. Section 3 discusses the simulation of the north-finding process at high speeds and a comparative analysis between the DNF-SCC algorithm and the existing cross-correlation (DNF-CC) algorithm. Furthermore, the design of an experimental FOG prototype and the experimental results of the DNF-SCC algorithm are discussed, validating the conclusions of the simulated experiments. Section 4 summarizes the key points of this paper.

## 2. Analysis Model of Dynamic North Finding

### 2.1. Dynamic North Finding Based on the Fiber Optic Gyroscope

**Figure 1:**
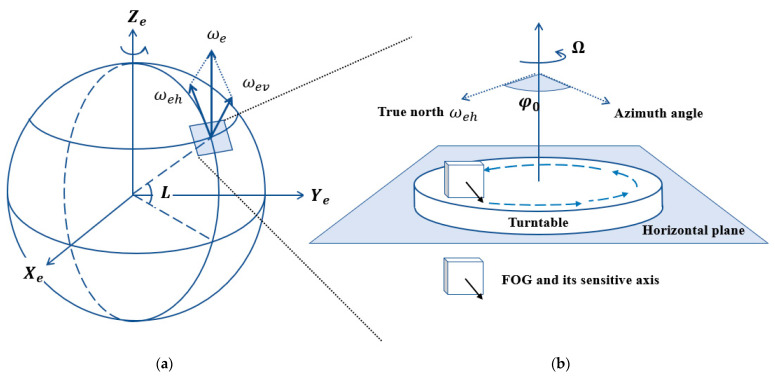
Principle of using a FOG to sense the Earth’s angular velocity: (**a**) stereogram view (**b**) side view.

A FOG can measure the angular rate around its sensitive axis. When mounted on a horizontal turntable with its sensitive axis parallel to the turntable, it can measure the horizontal component of the Earth’s rotational angular velocity as the turntable rotates. Figure 1 illustrates the principle of using a FOG to sense the Earth’s angular velocity. Let the angular velocity of Earth’s rotation at latitude L be $\omega_{e}$, which can be decomposed into a horizontal component $\omega_{eh}$ and a vertical component $\omega_{ev}$. The horizontal component $\omega_{eh}$ is parallel to the local horizontal plane and points north along the meridian line. According to the projection principle, $\omega_{eh}=\omega_{e}cosL$. The vertical component $\omega_{ev}$ is perpendicular to the local horizontal plane and points upward along the vertical line, and $\omega_{ev}=\omega_{e}sinL$.

When the sensitive axis of the FOG produces an angle $\varphi_{0}$ with the geographic north in the horizontal plane, the projection ω of the horizontal component $\omega_{eh}$ is calculated using:

(1) $$\omega =\omega_{eh}\cos\varphi_{0},$$

As the turntable drives the FOG to rotate at a constant speed Ω starting from the azimuth angle $\varphi_{0}$, at the position of the FOG at time t, the projection of the angular velocity of the Earth’s rotation can be expressed as follows:

(2) $$\omega (t)=\omega_{eh}\cos\left(\varphi_{0}+\Omega t\right).$$

The initial phase $\varphi_{0}$ of ω(t) is the desired azimuth angle.

### 2.2. Noises in the Dynamic North-Finding Process

#### 2.2.1. Self-Noise in Fiber Optic Gyroscope

The noise contained in the output of a FOG is a notable source of error. The error sources in a FOG can be categorized into three types [28,29]: scale factor error (K), bias $\omega_{0}$, and random noise $\varepsilon \left(t\right)$. Considering these errors, the output $\omega \left(t\right)$ of the FOG can be obtained as follows:

(3) $$\omega \left(t\right)=\omega_{0}+K\omega_{eh}cos\left(\varphi_{0}+\Omega t\right)+\varepsilon \left(t\right),$$

The scale factor, denoted as K, is affected by factors such as startup time and environmental temperature and can be calibrated in advance. When the north-finding time is generally short (less than 10 min), the scale factor undergoes relatively minor variations, inducing negligible influence on the north-finding accuracy. The bias $\omega_{0}$ represents the constant offset in the gyroscope output, and its impact can be eliminated through preliminary calibration [26].

The random noise $\varepsilon \left(t\right)$ inherent in the FOG can be isolated using the Allan variance analysis method. Originally devised to assess the frequency stability of timekeeping devices, the Allan deviation [30] proves applicable to the FOG due to their analogous characteristics [31]. For dynamic north finding, characterized by a sufficiently high sampling frequency, emphasis is placed on the angle random walk (ARW) and the rate random walk (RRW). The ARW affects the FOG’s short-term measurement accuracy, while the RRW influences its long-term accuracy [13]. Dynamic north-finding methods typically operate within short durations of less than 10 min. As instrumentation advances, the impact of the RRW on the FOG diminishes, and errors associated with the ARW become the primary factors influencing the dynamic north-finding results [32,33]. Therefore, the FOG’s output at any specified time ‘t’ can be expressed as the actual input angular rate, accounting for the influence of the ARW noise term. The noise term $\varepsilon \left(t\right)$ is represented as $\varepsilon \left(t\right)=\varepsilon_{ARW\left(t\right)}$. When Ω is expressed in units of (°/s), Equation (3) can be simplified as follows:

(4) $$\omega \left(t\right)=\omega_{0}+K\omega_{e}cosLcos\left(\varphi_{0}+2\pi \frac{\Omega }{360}t\right)+\varepsilon_{ARW\left(t\right)}.$$

#### 2.2.2. Turntable Speed Jitter

In various applications, the turntable’s core component is typically the servo motor. This motor employs a three-closed-loop proportional-integral-derivative control structure to precisely manage position and speed. The control strategy integrates a closed-loop feedback mechanism aimed at minimizing the deviation between the desired and transient position/velocity. While the deviation of speed continuously decreases during the control process, residual oscillation within a small range is maintained on the turntable’s rotation [21]. External environmental vibrations and voltage stability, however, may lead to sudden changes in speed. Although the turntable generally ensures relative speed accuracy within a specified range, high-speed north finding can amplify speed jitter by 10 to 100 times compared with low-speed conditions. This increase in speed jitter inevitably impacts the output of the FOG, affecting north-finding accuracy. Hence, in scenarios involving high turntable speeds during north finding, the influence of turntable speed jitter is pronounced and cannot be neglected.

Considering the continuous random jitter effect throughout the entire north-finding process, where the ideal turntable speed is Ω°/s, and the turntable speed jitter varies with time as ∆Ω(t), the output of the gyroscope $\hat{\omega }\left(t\right)$ can be expressed as:

(5) $$\hat{\omega }\left(t\right)=\omega_{0}+K\omega_{e}cosLcos\left[\varphi_{0}+2\pi \frac{\Omega +∆\Omega (t)}{360}t\right]+\varepsilon_{ARW\left(t\right)}.$$

As depicted in Equation (5), when the turntable speed remains stable ($∆\Omega \left(t\right)\sim 0$), the period of the signal $\hat{\omega }\left(t\right)$ remains unchanged. However, during turntable speed fluctuations, the frequency of $\hat{\omega }\left(t\right)$ changes. The greater the absolute error in the speed, the more significant the frequency variation in the $\hat{\omega }\left(t\right)$ signal. This change in pthe eriod directly influences the subsequent calculation of the north-finding value $\varphi_{0}$.

### 2.3. Dynamic North Finding Based on Segmented Cross-Correlation

#### 2.3.1. Traditional Cross-Correlation Algorithm

The cross-correlation method, a highly effective technique for analyzing weak sinusoidal signals [34] and extensively applied in seismology and acoustics [35], utilizes two orthogonal signals (sine and cosine) to calculate at the same frequency as the rotation frequency [36]. The traditional cross-correlation algorithm, denoted as DNF-CC, is a crucial method for the initial phase calculation in north-finding processes.

The cross-correlation method necessitates the use of two orthogonal signals, $y_{s}\left(t\right)$ and $y_{c}\left(t\right)$:

(6) $$y_{s}\left(t\right)=\sin\left(2\pi ft\right)\;,$$

(7) $$y_{c}\left(t\right)=\cos\left(2\pi ft\right)\;,$$

where f represents the rotation frequency of the FOG, determined with f=Ω/360.

The output $\hat{\omega }\left(t\right)$ of the FOG in Equation (5), when correlated with the functions with $y_{s}\left(t\right)$ and $y_{c}\left(t\right)$ at zero time lag (τ=0), respectively, can be expressed as follows [37]:

(8) $$R_{y_{s},\hat{\omega }}(\tau =0)\equiv R_{y_{s},\hat{\omega }}(0)=\frac{1}{T}\int_{0}^{t}y_{S}\left(t\right)\hat{\omega }\left(t\right)dt,$$

(9) $$R_{y_{c},\hat{\omega }}(\tau =0)\equiv R_{y_{c},\hat{\omega }}(0)=\frac{1}{T}\int_{0}^{t}y_{c}\left(t\right)\hat{\omega }\left(t\right)dt.$$

Equation (8) can be expressed as follows:

(10) $$R_{y_{s},\hat{\omega }}(0)=H+E_{1}+E_{2},$$

where

(11) $$H=\frac{1}{T}\int_{0}^{t}y_{S}\left(t\right)K\omega_{e}cosLcos\left[\varphi_{0}+2\pi \frac{\Omega +∆\Omega \left(t\right)}{360}t\right]dt,$$

(12) $$E_{1}=\frac{1}{T}\int_{0}^{t}y_{S}\left(t\right)\omega_{0}dt,\;$$

(13) $$E_{2}=\frac{1}{T}\int_{0}^{t}y_{S}\left(t\right)\varepsilon_{ARW\left(t\right)}dt.$$

For FOG data collected over an entire rotation cycle of the turntable, neglecting speed jitter, Equations (11)–(13) can be combined to derive the following equation:

(14) $$H=\frac{1}{T}\int_{0}^{t}\sin\left(2\pi ft\right)K\omega_{e}cosLcos\left(\varphi_{0}+2\pi ft\right)dt=-\frac{1}{2}K\omega_{e}cosLsin\varphi_{0},$$

(15) $$E_{1}=\frac{1}{T}\int_{0}^{t}y_{S}\left(t\right)\omega_{0}dt=0,$$

(16) $$E_{2}=\frac{1}{T}\int_{0}^{t}y_{S}\left(t\right)\varepsilon_{ARW\left(t\right)}dt=0.$$

$R_{y_{s},\hat{\omega }}$ can be simplified as follows:

(17) $$R_{y_{s},\hat{\omega }}\left(0\right)=-\frac{1}{2}K\omega_{e}cosLsin\varphi_{0}.$$

Similarly, $R_{y_{c},\hat{\omega }}$ can be simplified as:

(18) $$R_{y_{c},\hat{\omega }}(0)=\frac{1}{2}K\omega_{e}cosLcos\varphi_{0}.$$

The desired azimuth angle corresponding to the initial phase $\varphi_{0}$ of the FOG output signal $\hat{\omega }\left(t\right)$, can be calculated as follows:

(19) $$\varphi_{0}=\arctan\left(-\frac{R_{y_{s},\hat{\omega }}(0)}{R_{y_{c},\hat{\omega }}(0)}\right).$$

#### 2.3.2. Proposed Segmented Cross-Correlation Algorithm

**Figure 2:**
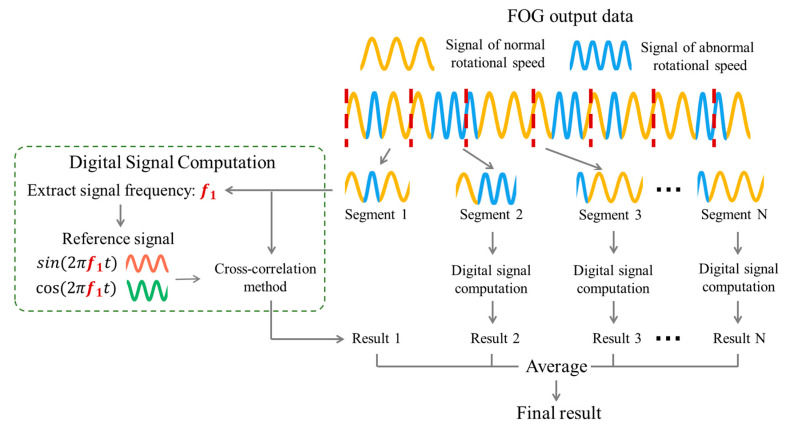
Proposed DNF-SCC flowchart.

The traditional DNF-CC algorithm assumes a constant turntable speed. However, in reality, the turntable speed varies over time due to speed jitter. Hence, when abnormal speed fluctuations occur frequently, this algorithm struggles to ensure north-finding accuracy. We propose a novel dynamic north-finding algorithm based on segmented cross-correlation (DNF-SCC) to suppress the impact of time-varying turntable speed by adopting a divide-and-conquer strategy. This algorithm mitigates the influence of speed fluctuations on north-seeking accuracy. Figure 2 illustrates the proposed north-finding algorithm flowchart. First, the output signal of the FOG under high turntable speeds is obtained, representing a multi-period cosine signal with noise. The yellow portion represents the FOG signal obtained at a normal turntable speed, whereas the blue portion represents the FOG signal with abnormal periods under irregular turntable speeds. Despite the high frequency of rotational speed fluctuations throughout the north-finding process, the turntable speed can be approximated as a constant value within short time intervals. Therefore, in the proposed DNF-SCC algorithm, the entire dataset is divided into multiple segments. Each segment undergoes digital signal computation to compute the azimuth value. Subsequently, the average value for each segment is calculated, yielding the final azimuth angle.

During the digital signal computation stage, the azimuth angle calculations employ the cross-correlation algorithm. The cross-correlation method requires generating a reference signal that matches the frequency of the measured signal. Considering the unpredictable influence of speed variations on signal frequency within signal segments, Fourier analysis is applied to each signal segment. In the spectrum of the segment signal, the frequency point with the maximum amplitude near the ideal frequency f is selected as the actual frequency of the segment. For the *n-th* segment ${\hat{\omega }}_{n}$, the corresponding frequency is expressed as $f_{n}$. Subsequently, the reference signals $y_{s,n}\left(t\right)$ and $y_{c,n}\left(t\right)$ are constructed:

(20) $$\left\{\begin{array}{c} y_{s,n}\left(t\right)=\sin\left(2\pi f_{n}t\right)\\ y_{c,n}\left(t\right)=\cos\left({2\pi f}_{n}t\right) \end{array}\right.\;.$$

The cross-correlation values $R_{y_{s},{\hat{\omega }}_{n}}$ and $R_{y_{c},{\hat{\omega }}_{n}}$ are calculated by applying the reference signals $y_{s,n}\left(t\right)$ and $y_{c,n}\left(t\right)$ to the FOG output signal.

(21) $$\left\{\begin{array}{c} R_{y_{s},{\hat{\omega }}_{n}}(0)=\frac{1}{T_{n}}\sum_{i=0}^{T_{n}-1}{\hat{\omega }}_{n}\left(i\right)y_{s,n}\left(i\right),i=1\ldots T_{n}\\ R_{y_{c},{\hat{\omega }}_{n}}(0)=\frac{1}{T_{n}}\sum_{i=0}^{T_{n}-1}{\hat{\omega }}_{n}\left(i\right)y_{c,n}\left(i\right),i=1\ldots T_{n} \end{array}\right.\;,$$

where $T_{n}$ represents the length of the *n-th* segment.

The initial phase $\varphi_{n}$ of the *n-th* segment can be calculated as:

(22) $$\varphi_{n}=\arctan\left(-\frac{R_{y_{s},{\hat{\omega }}_{n}}(0)}{R_{y_{c},{\hat{\omega }}_{n}}(0)}\right).$$

The final azimuth angle $\varphi_{0}$ is calculated by averaging the initial phase calculated by all segments:

(23) $$\varphi_{0}=\frac{1}{N}\sum_{n=1}^{N}\varphi_{n},$$

where N represents the number of segments.

The pseudocode for the DNF-SCC algorithm is presented as follows (Algorithm 1):
**Algorithm 1** Dynamic north-finding algorithm based on segmented cross-correlation (DNF-SCC)***Input:*** *FOG data, number of segments N.****Output:*** *Azimuth* *1.* *FOG data are divided into N segments* *2.* $\mathrm{An}\mathrm{array}v=\{v_{1},v_{2},\ldots ,v_{N}\}$*is initialized for recording the segment north-finding results* *3.* ***for*** *n = 1,2, …, N* *4.*          *FFT is used to obtain the frequency of the n-th segment* *5.*          *Reference signals are constructed based on the frequency of the n-th segment* ***6.***         *The north-finding value* $v_{n}$ *of the n-th segment is calculated according to the Equations (20)–(22)* ***7.*** ***end*** *8.* Azimuth=Averageofv *9.* ***return***  *Azimuth*

As can be seen from the pseudocode of the DNF-SCC algorithm, the algorithm complexity of DNF-SCC is O(NlogN), which is equivalent to that of the traditional DNF-CC algorithm.

## 3. Simulation and Experiment

### 3.1. Simulation

To validate the reliability of the proposed algorithm, we conducted simulations of the north-finding process using the FOG and mechanical turntables. The gyroscope’s output rate $f_{s}$ was set to 250 Hz, and we simulated the ARW noise of the FOG using white noise. Regarding the turntable rotational speed jitter, we simulated a scenario where the turntable speed suddenly changes. Initially, the rotational speed jitter parameters were set as follows: the amplitude of the turntable speed jitter was set to $9\times 10^{-4}{}^{\circ}/s$, and the probability of instability was set at 90%. The initial angle of the north-finding process was set to 15°, and the north-finding time was set to 360 s. To evaluate the algorithm’s performance at various turntable speeds, we simulated the north-finding results at speeds of 60°/s, 90°/s, 120°/s, and 180°/s.

**Figure 3:**
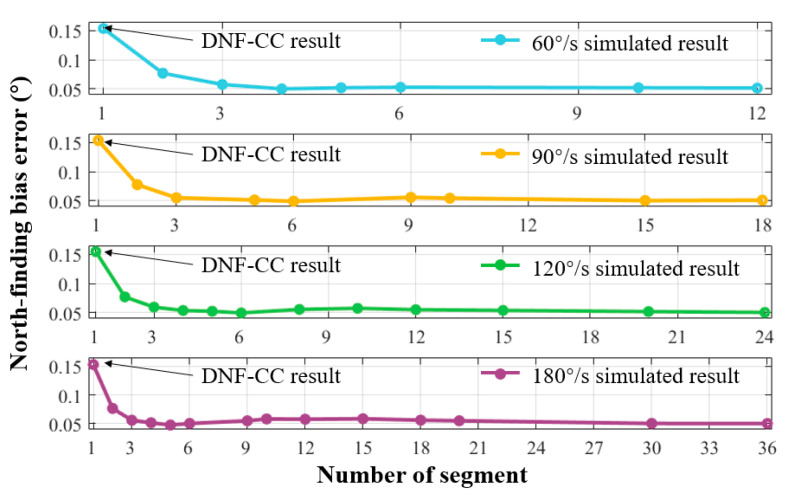
Simulation results indicating the relationship between north-finding bias error and the number of segments at various turntable speeds.

We divided the total number of cycles for each rotation speed into different segments over a north-finding interval of 360 s. Figure 3 illustrates the results of the calculations with different numbers of segments at various turntable speeds while maintaining a consistent north-finding time. The horizontal axis represents the number of segments, and the vertical axis indicates the bias error of the north-finding results. This bias error is calculated according to the average absolute deviation between the calculated azimuth and the true azimuth, reflecting the accuracy of north finding.

In the process of determining the frequency of the segment n with a length of m cycles using Fourier analysis, the frequency resolution $f_{res}$ should adhere to the following conditions:

(24) $$f_{res}=\frac{f_{s}}{T_{n}}=\frac{f_{s}}{\frac{360}{\Omega }mf_{x}-\xi }.$$

where $T_{n}$ represents the actual number of data points contained in segment n and *ξ* represents the difference between $T_{n}$ and the ideal scenario. An excessive number of segments may result in a short duration of data in each segment, leading to low-frequency resolution in the Fourier analysis. This would result in a decrease in the number of sampling points at the same turntable speed. At a minimum, the frequency resolution should be less than the rotation frequency corresponding to the turntable speed, that is, $f_{res}<\frac{\Omega }{360}$. Considering multiple cycles in the north-finding process, we adopted a minimum segment length of five cycles. When the number of segments is set to one, it includes all cycle data during the north-finding process, equivalent to the traditional cross-correlation algorithm. Hence, the first point in Figure 3 can be regarded as the outcome of the conventional dynamic north-finding algorithm using cross-correlation (DNF-CC).

Figure 3 illustrates that, across four distinct turntable speeds, the process of segmenting the data effectively reduces errors in north-finding precision. This phenomenon can be clarified by recognizing that the traditional DNF-CC algorithm involves constructing a reference signal with a single frequency and performing cross-correlation calculations with the entire dataset. However, when the turntable speed is unstable, the frequency of the FOG output fluctuates at different moments during the north-finding process. The DNF-CC algorithm struggles to rectify errors caused by random fluctuations in the turntable’s speed. In contrast, the proposed DNF-SCC algorithm, through the segmentation of data and extraction of the frequency of each segment separately, mitigates the impact of turntable speed jitter on the results.

With an increasing number of segments, the accuracy curves for each turntable speed exhibit minor, gradual fluctuations before reaching a minimum point, followed by a substantial increase. A combination of turntable speed jitter, FOG noise, and the number of segments influences this phenomenon. When the number of cycles within each segment is relatively small and the time within each segment is relatively short, the turntable speed fluctuations within each segment are relatively minor. However, during the cross-correlation calculation, the results are notably affected by the noise from the FOG. With the increasing number of windows, the noise of the FOG can be mitigated through averaging. As illustrated in Figure 3, for varying speeds, the north-finding bias error is minimal when the data is segmented into five to six sections. The north-finding bias errors are minimized to 0.0498°, 0.0492°, and 0.0496°, at speeds of 60°/s, 90°/s, and 120°/s, respectively. At a speed of 180°/s, the most superior north-finding result among the four tested speeds is achieved with a bis error of 0.0477°.

### 3.2. Experiment and Discussion

#### 3.2.1. Experimental Prototype

**Figure 4:**
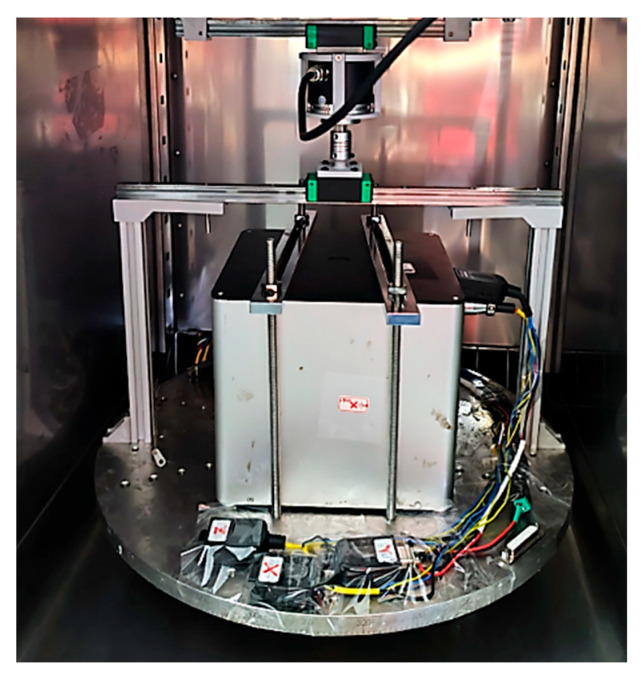
Experimental platform for the proposed DNF-SCC scheme.

The efficacy of the proposed algorithm underwent further validation through laboratory experiments. Figure 4 illustrates the experimental setup, which comprises a north finder equipped with a FOG, a turntable, and a photoelectric rotary encoder.

**Figure 5:**
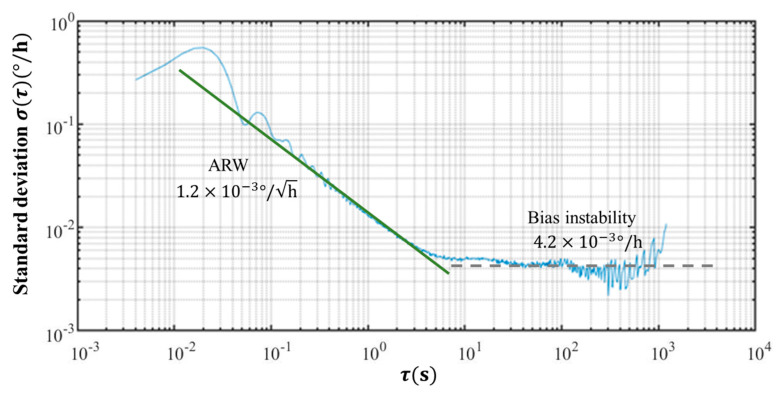
Measured Allan’s standard deviation of the FOG.

The noise performance of the FOG employed in this study was assessed through Allan deviation analysis. As Figure 5 shows, the ARW of the FOG was $1.2\times 10^{-3}{}^{\circ}/\sqrt{h}$, and the bias instability reach reached $4.2\times 10^{-3}{}^{\circ}/h$. The bias instability of the FOG was relatively small; hence we could consider the ARW the main noise source for the north-finding instrument during shorter north-finding times.

The FOG was securely mounted on the turntable and parallel to the horizontal plane with its sensitive axis. The entire assembly rotated on the local horizontal plane along with the turntable. Due to the insufficient output rate of the position parameter from the turntable’s internal control system, additional sensors were introduced to obtain real-time turntable position information with a higher output rate. A synchronized photoelectric rotary encoder recorded the rotation angle. When the turntable reached its initial position, the photoelectric rotary encoder displayed an identical value, enabling consistent data capture over the entire period at a constant rate.

#### 3.2.2. Experimental Result

The north-finding experiments were conducted at rotational speeds of 60°/s, 90°/s, 120°/s, and 180°/s using the same 360 s of north-finding time as in the simulation. Each north-finding experiment was repeated 10 times. Before the experiment, the high-precision static north-finding method and the low-speed dynamic method determined the true north direction, obtaining the reference azimuth. By comparing with the reference azimuth, bias errors in the north-finding results for the DNF-CC and DNF-SCC algorithms were obtained.

**Figure 6:**
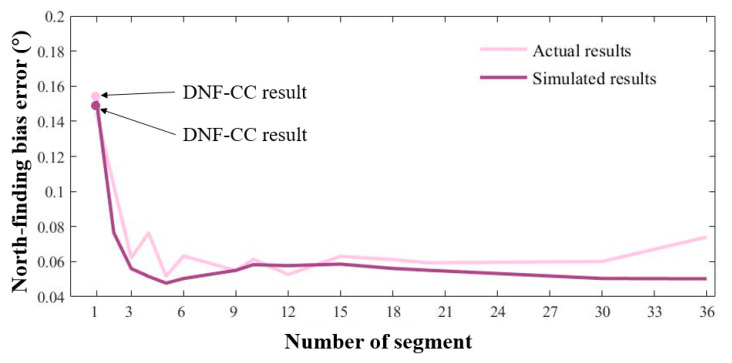
Comparison of the north-finding results between different numbers of segments in the model simulation and actual experimental results at a speed of 180°/s.

Figure 6 illustrates the comparison between the experimental results calculated using the DNF-SCC algorithm and the results in the simulations with a rotational speed of 180° and a north-finding time of 360 s. The simulation results are consistent with the experimental results. The error in north-finding accuracy is minimal when the number of data segments is five. As the data segmentation is reduced, the error in north-finding accuracy substantially increases.

**Figure 7:**
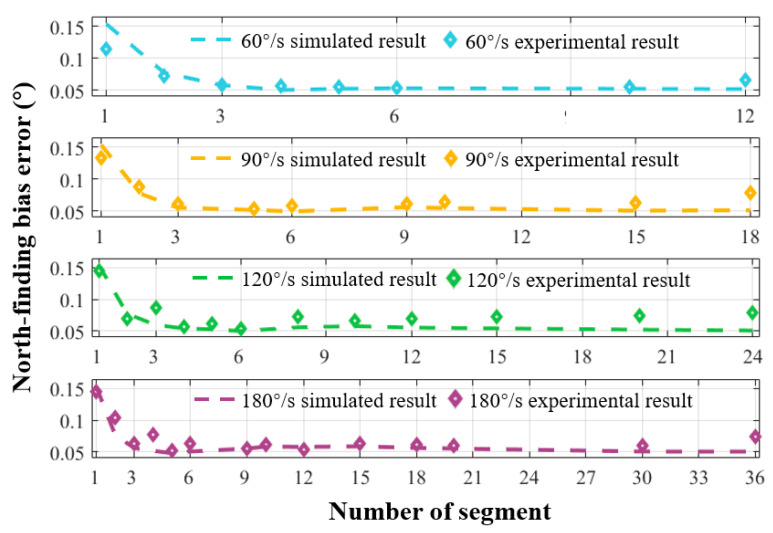
Experimental results of the relationship between north-finding accuracy error and segment number across various turntable speeds.

Figure 7 depicts the experimental results between the north-finding accuracy and segment length at turntable speeds of 60°/s, 90°/s, 120°/s, and 180°/s. The simulation results are consistent with the experimental results, confirming the validity of the proposed error model. The results also indicate that the accuracy of the DNF-SCC algorithm surpasses that of the DNF-CC approach (data points with the segment number of one). Optimal north-finding results are achieved when the data for each turntable speed is divided into five to six segments, with fluctuations between the measured results and the simulation results due to the instability of random vibrations in the experimental environment.

**Figure 8:**
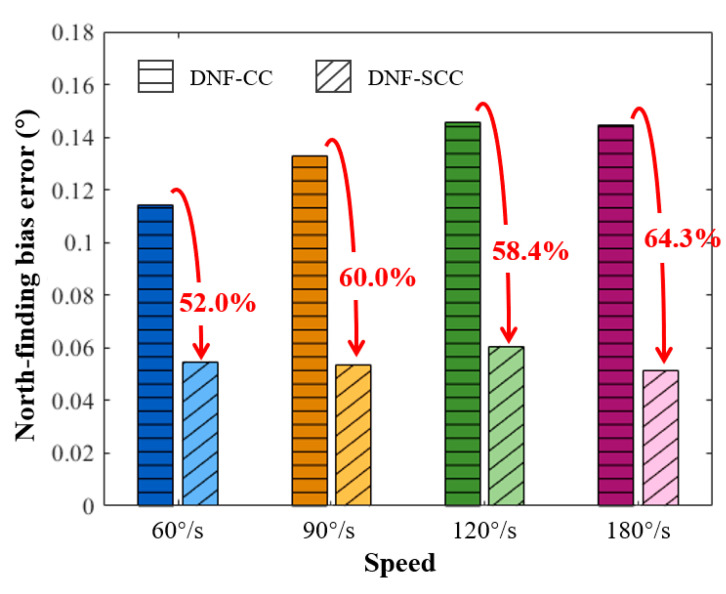
Comparison results between the proposed DNF-SCC algorithm and traditional DNF-CC algorithm at various turntable speeds.

To compare the north-finding performance of the proposed DNF-SCC algorithm with the conventional DNF-CC algorithm, we segmented the 360 s data into five segments at various turntable speeds. The azimuth angle was then calculated using both the DNF-SCC and DNF-CC algorithms. Figure 8 illustrates the bias errors obtained from the results of the DNF-SCC and DNF-CC algorithms. As shown in Figure 8, the utilization of the DNF-CC algorithm leads to a persistent north-finding bias error exceeding 0.1° at different speeds due to the random jitter in turntable speed. Moreover, with increasing turntable speed, the bias error of the DNF-CC algorithm exhibits an upward trend, indicating a decline in north-finding performance at higher speeds. By contrast, the proposed DNF-SCC algorithm significantly enhances north-finding performance. At turntable speeds of 60°/s or higher, the north-finding error of the DNF-SCC algorithm consistently remained below 0.061°, generally more than 50% lower than the results obtained with the traditional DNF-CC algorithm. Notably, at a speed of 180°/s, the north-finding bias error of the DNF-SCC algorithm was 0.052°, significantly lower than that of the DNF-CC algorithm, representing an improvement of over 64%. These results highlight the superior ability of the DNF-SCC algorithm to maintain high north-finding accuracy at elevated turntable speeds.

#### 3.2.3. Discussion

While our constructed model demonstrates good agreement with the actual data, differences persist between the simulation’s results and real-world conditions. The observation from Figure 3 indicates that the simulation results, calculated using the DNF-CC algorithm, do not exhibit a significant decrease with increasing turntable speed. The reason for this phenomenon is that accurately modeling the speed errors of the turntable becomes a significant challenge without comprehensive information on its mechanical structure. In contrast to the DNF-SCC algorithm, the DNF-CC algorithm employs complete data for calculations and necessitates the consideration of more intricate processes, potentially leading to greater errors in the simulation results. Nevertheless, the numerical simulation method still maintains trend agreement with the actual results, demonstrating the potential advantages of segmented data processing. In the future, we plan to enhance our modeling approach by incorporating the specific mechanical characteristics of the turntable. This would allow for a more refined representation of the rotation process.

In this paper, the segmentation method employed using the DNF-SCC algorithm is based on the simple average segmentation principle. We recognize that this approach is only a preliminary exploration and that future research will focus on developing more advanced north-finding strategies. Specifically, we will investigate the impacts of dividing data into varying segment lengths and explore the methods of assigning different weights to different segments of data. Through these studies, we anticipate achieving enhanced north-finding performance.

## 4. Conclusions

This paper introduces the DNF-SCC algorithm, a dynamic north-finding algorithm for FOGs designed to mitigate turntable jitter. Unlike traditional algorithms that assume a constant turntable speed for calculations, our proposed DNF-SCC algorithm employs a divide-and-conquer strategy. This approach alleviates the impact of time-varying turntable speed, effectively suppressing the effects of speed jitter on north-finding accuracy. The algorithm adeptly tackles challenges associated with variable turntable speeds, enhancing north-seeking precision in practical applications. The algorithm’s effectiveness was assessed through comprehensive simulations and experiments encompassing various speeds and segment lengths. The results from both the simulations and experiments affirm the capability of the DNF-SCC algorithm in elevating north-finding accuracy. Notably, at a turntable speed of 180°/s, the DNF-SCC algorithm achieved a north-finding error of 0.052°, marking a remarkable improvement of over 64% compared with traditional DNF-SCC algorithms at high turntable speeds. These findings underscore the practical advantages offered by the proposed DNF-SCC algorithm, presenting a reliable solution for dynamic north-finding applications in FOGs, especially when a rapid north-finding response is crucial.

## Data Availability

Data are contained within the article.
